# Astaxanthin alleviates fipronil-induced neuronal damages in male rats through modulating oxidative stress, apoptosis, and inflammatory markers

**DOI:** 10.1038/s41598-025-95447-3

**Published:** 2025-04-24

**Authors:** Mona H. Hafez, Ali H. El-Far, Samar S. Elblehi

**Affiliations:** 1 Department of Physiology, Faculty of Veterinary Medicine, Alexandria University, Alexandria, 22758 Egypt; 2 Department of Biochemistry, Faculty of Veterinary Medicine, Damanhour University, Damanhour, 22511 Egypt; 3 Department of Pathology, Faculty of Veterinary Medicine, Alexandria University, Alexandria, 22758 Egypt

**Keywords:** Astaxanthin, Fipronil, Oxidative stress, Neurotransmitters, Proinflammatory cytokines, Immunohistochemistry, Biochemistry, Cell biology, Neuroscience, Physiology, Neurology

## Abstract

Fipronil (FPN) is an effective pesticide for veterinary and agricultural use; however, it can induce neurotoxic effects on non-target organisms after accidental exposure. Astaxanthin (AST) is a dark red carotenoid with antioxidant, anti-inflammatory, neuroprotective, and antiapoptotic effects. This study investigated the ameliorative impact of AST against FPN-induced brain damage in rats. Thirty-two adult Wistar rats were allocated into four groups (*n* = 8): Control, AST (20 mg/kg bwt/day), fipronil (FPN) (20 mg/kg bwt/day), and AST + FPN group. Acetylcholine (ACh), dopamine, malondialdehyde (MDA), and proinflammatory cytokines, including tumor necrosis factor-α (TNF-*α*), interleukin-1β (IL-1*β*), interleukin-6 (IL-6), and inflammatory cytokine cyclooxygenase-2 (COX2) levels were enhanced in the FPN-administered group relative to the control group. In addition, a substantial reduction of acetylcholine esterase (AchE), gamma-aminobutyric acid (GABA), serotonin, reduced glutathione (GSH) levels, catalase (CAT), and total superoxide dismutase (T-SOD) enzyme activities were determined. FPN induced histopathological alterations in the cerebral and cerebellar tissues. Likewise, the histomorphometric image analysis of H and E-stained tissue sections was constant with FPN-induced neurotoxicity. Immunohistochemically, an intense positive immunohistochemical staining of apoptotic marker caspase-3 and astrocytes activation marker glial fibrillary acidic protein (GFAP) in the examined tissues was noticed. Inversely, the simultaneous administration of AST partially attenuated FPN impacts, ameliorating the severity of FPN-induced neuronal damage. These results were also established with the molecular docking findings. It could be suggested that AST has antioxidant, anti-inflammatory, and anti-apoptotic capabilities against FPN-induced neuronal damage via suppression of oxidative stress and pro-inflammatory cytokines, preservation of the neurotransmitters, and the cerebral and cerebellar histoarchitectures.

## Introduction

Repeated exposure to environmental pollutants such as pesticides can lead to neurotoxicity in humans, animals, and rodents^1^ and negatively impact brain functions and histoarchitecture^2–4^. In public health, pesticides eliminate disease-carrying organisms, such as agricultural pests^5^. Due to their potential hazards to humans and other living organisms, pesticides must be applied cautiously and disposed of following proper safety protocols. Fipronil (FPN,4-trifluoromethylsulfinylpyrazole-3-arbonitrile) is a phenylpyrazole broad-spectrum insecticide categorized as a class II moderately dangerous pesticide by the World Health Organization (WHO)^6^. It is commonly consumed in agriculture, horticulture, and veterinary practices^7^. Understanding the neurological effects of FPN is essential since both animals and humans may experience chronic low-dose exposure or accidental high doses. Inside the insects’ central nervous systems, FPN blocks gamma-aminobutyric acid (GABA) chloride channels, effectively targeting those resistant to other insecticides^3,7^. FPN can cause neuronal dysfunctions in mammals by binding firmly to GABA chloride channels, which leads to hyperexcitability. Its primary metabolite, fipronil sulfone, has an even higher affinity for mammalian GABA receptors, indicating that FPN’s metabolites may harm non-target animals^3^. GABAergic interneurons linking the entorhinal cortex and hippocampus are crucial for spatial and perceptual memory. Their suppression in the prefrontal cortex delays cognitive activities^8^. It was also reported that in FPN-administered rats, serotonin levels decreased in the striatum, hippocampus, and hypothalamus^7^.

Numerous research studies indicated that FPN may disrupt the body’s natural antioxidant defense system, generating reactive oxygen species (ROS) and reducing antioxidant reserves in target cells^9,10^. Vascular changes such as congestion, ischemia, hypoxia, lipid peroxidation, and DNA damage are believed to result from these events^6^. Alterations in a cell’s redox balance often led to mitochondrial dysfunction, ultimately triggering apoptosis^9^. It is thought that FPN stimulates the production of ROS, which releases inflammatory and apoptotic markers, leading to neuronal cell death^11^. Proteins, lipids, and DNA are critical cellular macromolecules damaged by excessive ROS, causing cell dysfunction and disruption^12^. It was also reported that FPN inhibits the state 3 respiration in mitochondria energized with glutamate plus malate, substrates of complex I of the respiratory chain, and decreases the mitochondrial membrane potential, inhibiting ATP synthesis^13^.

Neuroinflammation and apoptosis are common causes of FPN-induced neuronal damage, indicated by caspase-3 activation, glial fibrillary acidic protein (GFAP) upregulation, inducible nitric oxide synthase overexpression, and neuronal degeneration^2,14^. In the SH-SY5Y cell line, FPN inhibits the energy supply, leading to mitochondrial malfunction and ATP depletion. These events could trigger apoptotic cell death by activation of caspase-3 signaling^15^.

Astaxanthin (AST; 3,3-dihydroxy-*β*, *β*-carotene-4,4-dione) is a lutein carotenoid in many micro- and marine species, including shrimp, crayfish, salmon, yeast, and trout. The hydroxyl groups in AST’s *ββ*-ionone rings are connected to two asymmetric carbons (3 and 3′)^16,17^. Because of its hydrophobic structure, which comprises terminal polar groups and a conjugated polyene, AST can pass through cell membranes and enter subcellular spaces^18^. AST impacts most organs and tissues’ biochemical activities^19,20^. AST, located in the cell membrane, scavenges free radicals, preserves membrane structure, enhances immune performance, and regulates gene expression, protecting cells from oxidative damage and maintaining tissue integrity^21,22^. AST, amid many carotenoids, has anti-inflammatory, antioxidant, anti-apoptotic, antiproliferative, neuroprotective, anti-diabetic, and possesses eye-, skin-, reno-, and hepato-protective abilities^23^. Numerous experiments showed that AST boosted the brain’s nuclear factor erythroid 2-related factor-2 (Nrf2)/antioxidant response elements system, directly reducing oxidative stress and indirectly lessening oxidative damage^24^. Furthermore, AST demonstrated multipotent biological properties, including enhanced immunity,  anti-tumor, anti-inflammatory, and anti-apoptotic properties^17^. AST effectively combated apoptosis by blocking crucial components such as cytochrome c, caspase-3, and caspase-9 while modulating the Bcl-2  associated X-protein/BCL2 apoptosis regulator ratio. This decisive action underscores its potential to promote cell survival^25^. Furthermore, AST can cross the blood–brain barrier (BBB) and has no adverse side effects^18^. It has the potential to ameliorate brain damage in numerous neurological disorders such as dopaminergic neurodegeneration, Parkinson’s disease^26^traumatic brain injury, and cognitive impairment in Alzheimer’s disease^20^.

The current work was aimed to assess, for the first time, the possible ameliorative impact of AST against FPN-induced oxidative stress, inflammation, neurotransmitter disruption, histopathological changes, apoptosis, and astrogliosis in the brain tissues of male Wistar rats.

## Materials and methods

### Chemicals and reagents

AST was attained from Carbosynth Limited, UK, Code: FA18001), while dimethyl sulfoxide (DMSO) (0.25% v/v) was bought from SNL chem Co. FPN (Coash SC 20%), a preparation from Star Chem. Company (Wellford, SC, USA) and manufactured by Zhejiang Yongnong Chem. Co. (Shaoxing, China). Rat’s neurotransmitters, including acetylcholine (Ach), acetylcholinesterase (AchE), GABA, serotonin or 5-hydroxytryptamine (5-HT), and dopamine ELISA kits were obtained from Chongqing Biospes Co. Ltd. (Chongqing Shi, China).

Malondialdehyde (MDA), total superoxide dismutase (T-SOD), reduced glutathione (GSH), and catalase (CAT) kits were purchased from Biodiagnostic (Cat. Tahrir, Cairo, Egypt). Proinflammatory cytokines, including interleukin-6 (IL-6), tumor necrosis factor-α (TNF-*α*), interleukin-1*β* (IL-1*β*), and inflammatory cytokine cyclooxygenase-2 (COX2) ELISA kits were attained from Anogen, Mississauga, Ontario, Canada. Rabbit polyclonal anti-cleaved caspase-3 antibody (AB3623) was obtained from Merck Millipore (Darmstadt, Germany), and rabbit polyclonal anti-GFAP antibody (ab7260) was bought from Abcam (Cambridge, UK). 3,3-diaminobenzidine tetrahydrochloride Kit (DAB) obtained from Thermo Fisher Scientific (Rockford, IL, USA). All chemicals used were of analytical grade and were consumed as received, lacking any additional purification.

### Ethics statement

The Animal Care Review Committee of the Faculty of Veterinary Medicine at Alexandria University, Egypt, approved all experiments under the Ethical Committee Approval number of 2023/013/263. We followed the United States National Academy of Sciences’ "Guide for the Care and Use of Laboratory Animals, ensuring humane treatment and minimizing animal suffering.

### Animals

Thirty-two male Wistar rats weighing 180–200 g and 7 weeks of age were used in this investigation. The rats were housed in plastic cages that allowed them unlimited access to water and a typical lab diet. Throughout the trials, rats were maintained at 22—25 °C in the natural room temperature range with a light cycle and humidity. Rats were obtained from the Medical Research Institute at Alexandria University in Egypt and were kept for two weeks to acclimate to the laboratory environment.

### Experimental groups

Rats were randomly assigned into four equal groups of eight rats in each group as follows:

i. The rats in group 1 (control group) were intra-gastrically administered with 0.5 ml of DMSO (0.25% v/v) by oral gavage.
ii. The rats in group 2 (AST group) received AST (20 mg/kg bwt /day) dissolved in DMSO (0.25% v/v)^21^ for four weeks by oral gavage. The dose was adjusted weekly to account for variations in body weight, ensuring a consistent dose for each kilogram of the rat’s body weight throughout the entire experimental period.
iii. The rats in group 3 (FPN group) received FPN (20 mg/kg bwt /day) orally for 4 weeks^27^.
iv. The rats in group 4 (AST + FPN group) were treated as groups 2 and 3.

### Blood and tissue collection and preparation

Blood samples were taken straight from the heart in a clean dry tube at room temperature 24 h after the last previous dosing under ketamine/xylazine anesthesia (7.5 and 1.0 mg/kg ip). The samples were then centrifuged at 1,800 × g for 15 min to separate serum and investigate proinflammatory cytokines (IL-6, IL-1*β*, and TNF-*α*) and inflammatory cytokines (COX2). Following the collection of blood, and while the animals were still anesthetized, euthanasia was performed via decapitation and their brains were meticulously removed from their skulls and examined closely.

Each rat’s brain tissue was rinsed with physiological saline (NaCl 0.9%) and then cut into two halves. The left half was rinsed with deionized water, blotted dry, and perfused with a 50 mM sodium phosphate buffer (pH 7.4) comprising 0.1 mM ethylenediaminetetraacetic acid (EDTA) to remove red blood cells and platelets. The tissues were homogenized in ice-cold buffer, centrifuged for 30 min at 10,000 × g, and the supernatant was kept at -80°C for neurotransmitter (ACh, AchE, GABA, 5-HT, and dopamine), oxidative stress markers (MDA), and antioxidant status (GSH, CAT, and T-SOD) determinations. The right halves were kept in 10% neutral-buffered formalin for at least 24 h for further histopathological and immunohistochemical studies.

### Neurotransmitters and their catabolizing enzyme determination

The brain contents of 5-HT, ACh, and dopamine were analyzed following the manufacturer’s instructions using 5-HT rat ELISA kit (catalog no. BYEK2838), ACh rat ELISA kit (catalog no. BYEK2989), and dopamine rat ELISA kit (catalog no. BYEK2898), respectively, from Chongqing Biospes Co. Ltd. The enzymatic activity of AchE in brain tissue homogenates was evaluated by the colorimetric technique of Ellman et al.^28^. GABA was evaluated by measurable HPLC utilizing the precolumn phenyl isothiocyanate (PITC, Edman’s Reagent) derivatization procedure method^29^.

### Quantification of neural lipid peroxidation and antioxidants

The brain tissue homogenates were used for MDA^30^ and GSH^31^ levels and CAT^32^ and T-SOD^33^ activities determination. Protein levels in brain tissues were determined following the method of Lowry et al.^34^.

### Examination of proinflammatory and anti-inflammatory cytokines

Proinflammatory cytokines (IL-6, TNF-*α*, and IL-1*β*) and inflammatory cytokine (COX2) were determined in serum following the manufacturer’s protocols of purchased ELISA kits^35^. All analyses were confirmed by an ELISA Plate Reader (Bio-Rad, Hercules, CA, USA).

### Histopathological examination and lesion scoring

Following necropsy, brain tissue specimens (cerebrum and cerebellum) were obtained from different rat groups (*n* = 8 per group), rinsed in physiological saline solution (NaCl 0.9%), and then placed at least for 24 h in 10% neutral buffered formalin (pH 7.4). Fixed tissue specimens have been processed using the conventional paraffin embedding technique, sectioned at four µm, and stained with Mayer’s hematoxylin and eosin (H&E) stain following the method described by Bancroft et al.^36^. Stained tissue sections were inspected with a light microscope (Leica DM500). Photomicrographs were captured using a digital camera (Leica EC3, Leica, GmbH, Wetzlar, Germany).

The degree of severity of the observed histopathological findings was represented using a four-point semi-quantitative scoring system^37^ as follows: normal histology (-), mild ( +), moderate (+ +) and severe (+ + +) damage. Eight slides from eight different rats per group were evaluated to assess and grade the varied pathological alterations observed in the studied brain tissues. Each rat’s score was determined based on the area of the same slide. In addition, the grade was determined by calculating the median score for each group.

### Histomorphometrical analysis

The quantitative analysis of the cerebral and cerebellar tissues was performed using Image J analysis software (Image J v1.46r, National Institute of Health, Bethesda, MD, USA). Images of H&E-stained cerebral cortex and cerebellum sections were used to quantify the number of different cell types using the cell counter plug-in available on Image J (manual computer-assisted cell counting, ImageJ plug-in-cell counter.jar). After applying a grid across the image, the number of various cell types in the respective brain regions was counted. To prevent bias, these morphometric measurements were conducted blindly for images of 10 fields per section for 8 rats per group (HPF, × 400).

### Immunohistochemical studies and quantitative assessment

The localization of cleaved caspase-3 and GFAP was identified using immunohistochemical labeling^38^. The cerebral and cerebellar paraffin tissue sections of four µm thickness were taken on positively charged slides, then deparaffinized in xylene and rehydrated in descending ethanol concentrations. Microwave-assisted antigen retrieval was done to expose the antigen by boiling the slides with 10 mM citrate buffer, pH 6.0, for 10 min, then cooling for 20 min at room temperature. After washing with phosphate buffer saline, endogenous peroxidase was inactivated using 3% hydrogen peroxide in absolute methanol for 5 min at room temperature. Then, the non-specific reaction was blocked by incubating the slides with 2% bovine serum albumin in TBS for 60 min. The tissue sections were incubated overnight at 4 °C with rabbit polyclonal anti-cleaved caspase-3 antibody (1:100, AB3623 Merck Millipore, Darmstadt, Germany) and rabbit polyclonal anti-GFAP antibody (1:200, ab7260, Abcam, Cambridge, UK). After washing three times with Dako Tris-buffered saline, the tissue slices were treated with biotinylated goat anti-rabbit IgG antibody (AB132; Sigma Aldrich, Saint Louis, USA) for 30 min at 37 °C followed by incubation of the sections with the streptavidin-conjugated horseradish peroxidase reagent (18–152 Millipore, Merck, Darmstadt, Germany) and for 60 min at 37° C after being rinsed in Tris-buffered saline. To initiate a peroxidase reaction, the sections were rinsed with a washing buffer and subsequently incubated with 3,3-diaminobenzidine tetrahydrochloride (DAB, Thermo Fisher Scientific, Rockford, IL, USA). The tissue sections were counterstained with Mayer’s hematoxylin to improve nuclear staining, then cleared in xylene, dehydrated in absolute alcohol, and mounted with di-poly cysteine xylene (DPX). Immunoreactivity was visualized as dark brown staining of cleaved caspase-3 (brown coloration of nerve cells) and GFAP (brown coloration of the astrocytes, including their bodies and processes) using a light microscope. Micrographs of ten random fields from each section were captured at a magnification power of 400 × using a digital camera (EC3, Leica, Germany) linked to a Leica microscope (DM500) to quantify the immunoreactivity. ImageJ software (ImageJ software, v1.46r, National Institutes of Health, Bethesda, MD USA) was used to analyze these images and to estimate the area percentage (%) of cleaved caspase-3 and GFAP-positive brown immune-stained cells according to Schneider et al.^39^.

### Molecular docking

The three-dimensional (3D) structures of caspase-3 (ID: P55213), AchE (ID: P37136), superoxide dismutase-1 (SOD1; ID: P07632), SOD2 (ID: P07895), and CAT (ID: P04762) proteins were obtained from the UniProt (https://www.uniprot.org/) database. The 3D structures of FPN (ID: 3352) and AST (ID: 5,281,224) were retrieved from the PubChem database (https://pubchem.ncbi.nlm.nih.gov/).

The 3D structures of protein and ligands were prepared for docking with energy minimization using Chimera 1.16 software^40^ while molecular docking was performed using InstaDock software^41^, and the molecular interactions between them were visualized by BIOVIA Discovery Studio Visualizer 2016 software.

### Statistical analysis

GraphPad Prism v.9 (https://www.graphpad.com/) (GraphPad, San Diego, CA, USA) analyzed the data by a one-way ANOVA with Tukey’s post hoc multiple range testing. Data were presented as mean ± SD. All statements of significance were at *P* < 0.05*.*

## Results

### Neuronal neurotransmitters profile

**Fig. 1:**
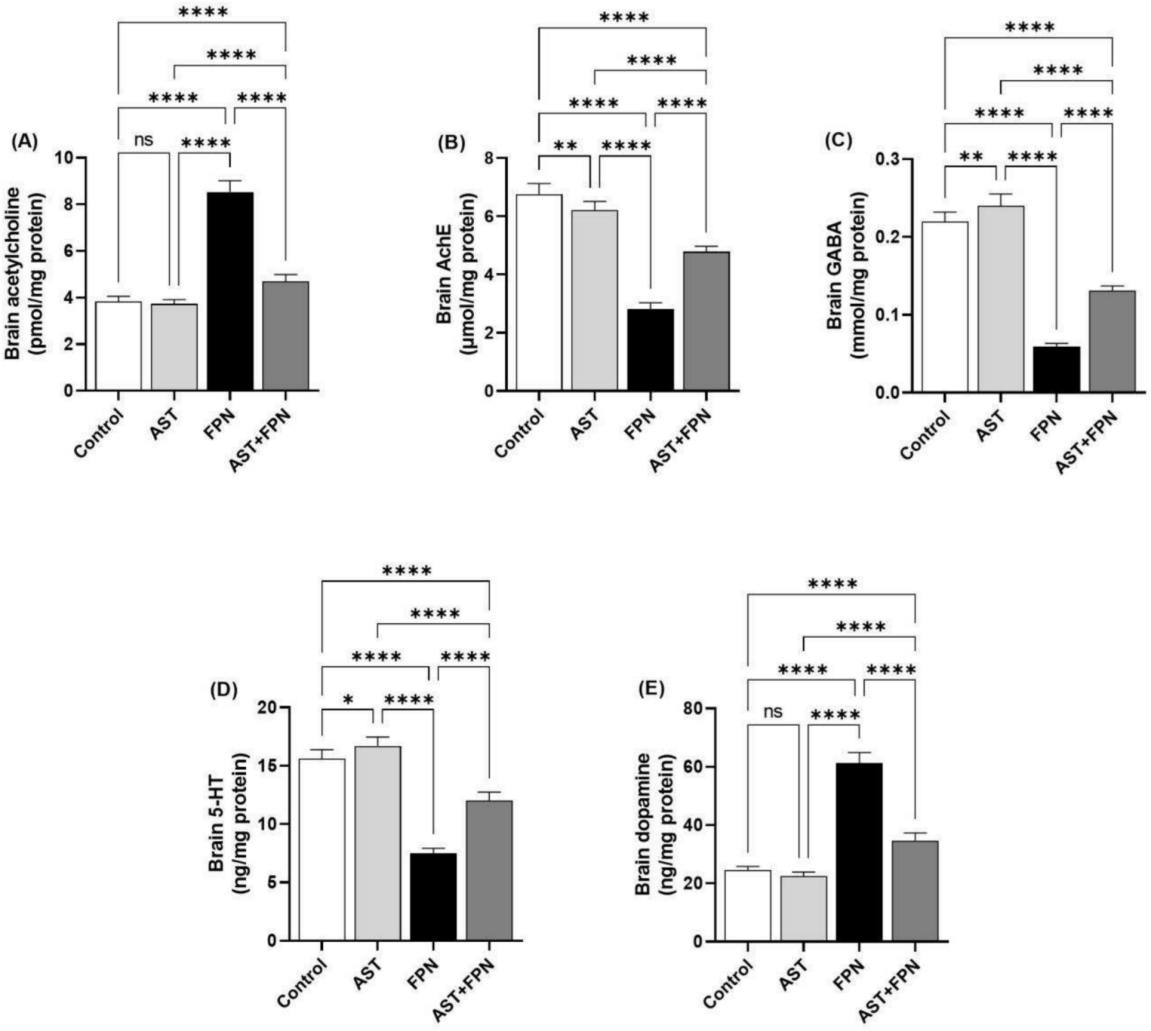
Effect of Astaxanthin (AST) (20 mg/kg bwt) on brain neurotransmitters profile of fipronil (FPN) (20 mg/kg bwt) administered male rats. (**A**) Acetylcholine. (**B**) Acetylcholine esterase (AchE). (**C**) Gamma-aminobutyric acid (GABA). (**D**) 5-hydroxytryptamine (5-HT = serotonin). (**E**) Dopamine. Data were analyzed with a one-way ANOVA followed by Tukey’s multiple comparison test. Data are expressed as the mean ± SD; n = 8. Means within columns carrying * are significantly different at *P* < 0.05, **P < 0.01, and ****P < 0.0001. ns = nonsignificant.

Our results showed that FPN caused a substantial (*P* < 0.001) enhancement in brain Ach and dopamine concentrations, in addition to a marked decrease in values of AchE, GABA, and serotonin relative to the control group (Fig. 1). AST + FPN treatment attenuated these effects by significantly (*P* < 0.001) reducing the neuronal Ach and dopamine and enhancing the AchE, GABA, and serotonin levels relative to the FPN group. A non-significant dissimilarity of Ach and dopamine levels was observed between the AST and the control groups; interestingly, a marked increase (*P* < 0.01) of the brain’s AchE, GABA, and serotonin levels was observed in the AST-only administered group relative to the control one.

### Brain oxidant-antioxidant status

**Fig. 2:**
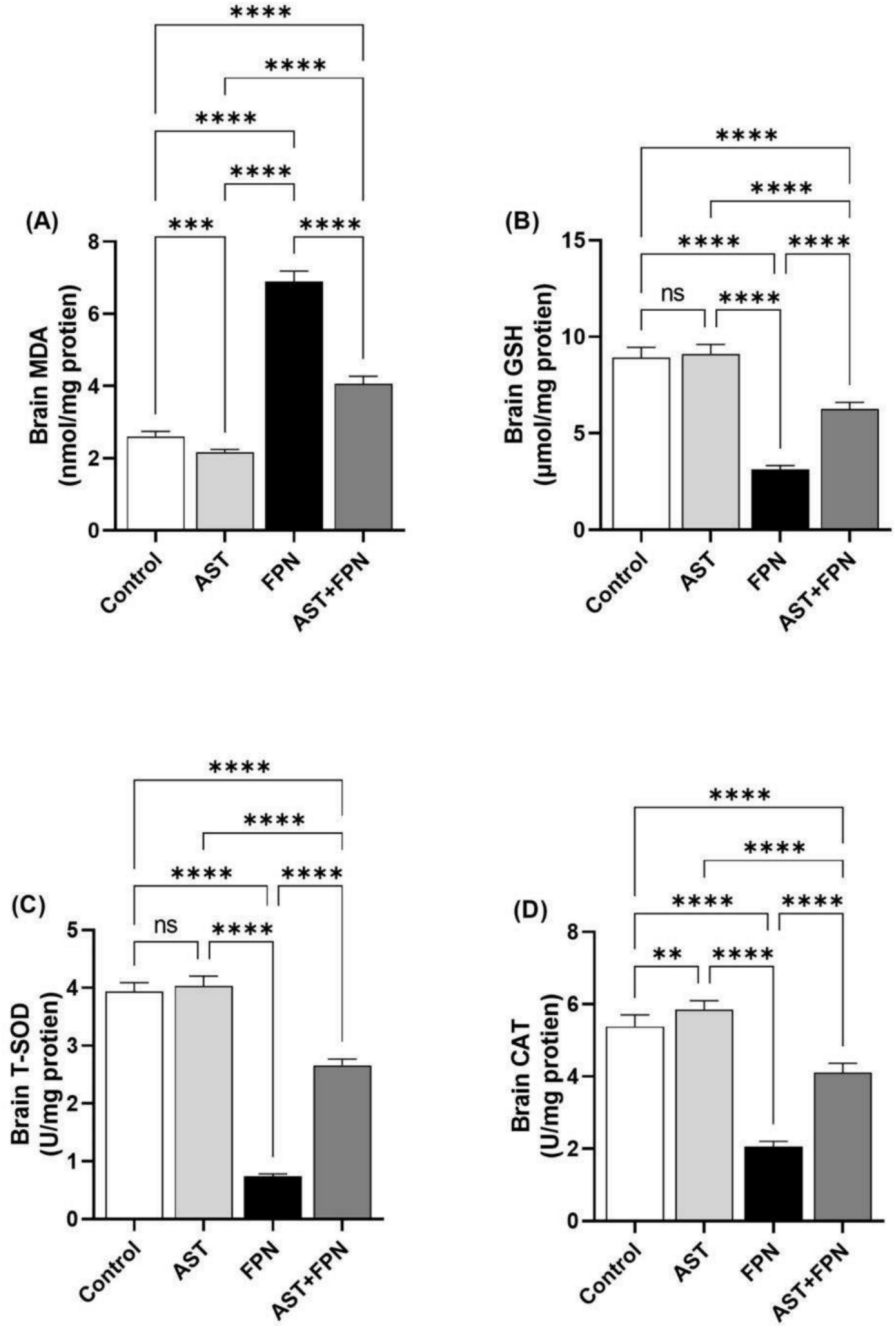
Effect of Astaxanthin (AST) (20 mg/kg bwt) on brain oxidant-antioxidant status of fipronil (FPN) (20 mg/kg bwt) administered male rats. (**A**) Malondialdehydes (MDA). (**B**) Reduced glutathione (GSH). (**C**) Total superoxide dismutase (T-SOD). (**D**) Catalase (CAT). Data were analyzed with a one-way ANOVA followed by Tukey’s multiple comparison test. Data are expressed as the mean ± SD; n = 8. Means within columns carrying ** are significantly different at *P* < 0.01, ***P < 0.001, and ****P < 0.0001. ns = nonsignificant.

The FPN-administered rats revealed a marked (*P* < 0.001) increase in MDA concentration associated with a significant decrease in T-SOD, GSH, and CAT activities compared with the control group. Nevertheless, AST treatment reduced the concentration of increased MDA relative to the FPN group; conversely, the antioxidant biomarkers were partially reestablished in the AST + FPN-treated rats relative to the FPN control (Fig. 2). Interestingly, MDA levels were significantly decreased (*P* < 0. 0.001) in AST-administrated rats relative to the control group; also, CAT activity was improved markedly (*P* < 0.0.01) in the AST-supplemented group compared with the control group. A non-significant difference in the brain’s GSH and T-SOD levels was recorded between the AST-supplemented and control groups.

### Serum pro-inflammatory and inflammatory cytokines

**Fig. 3:**
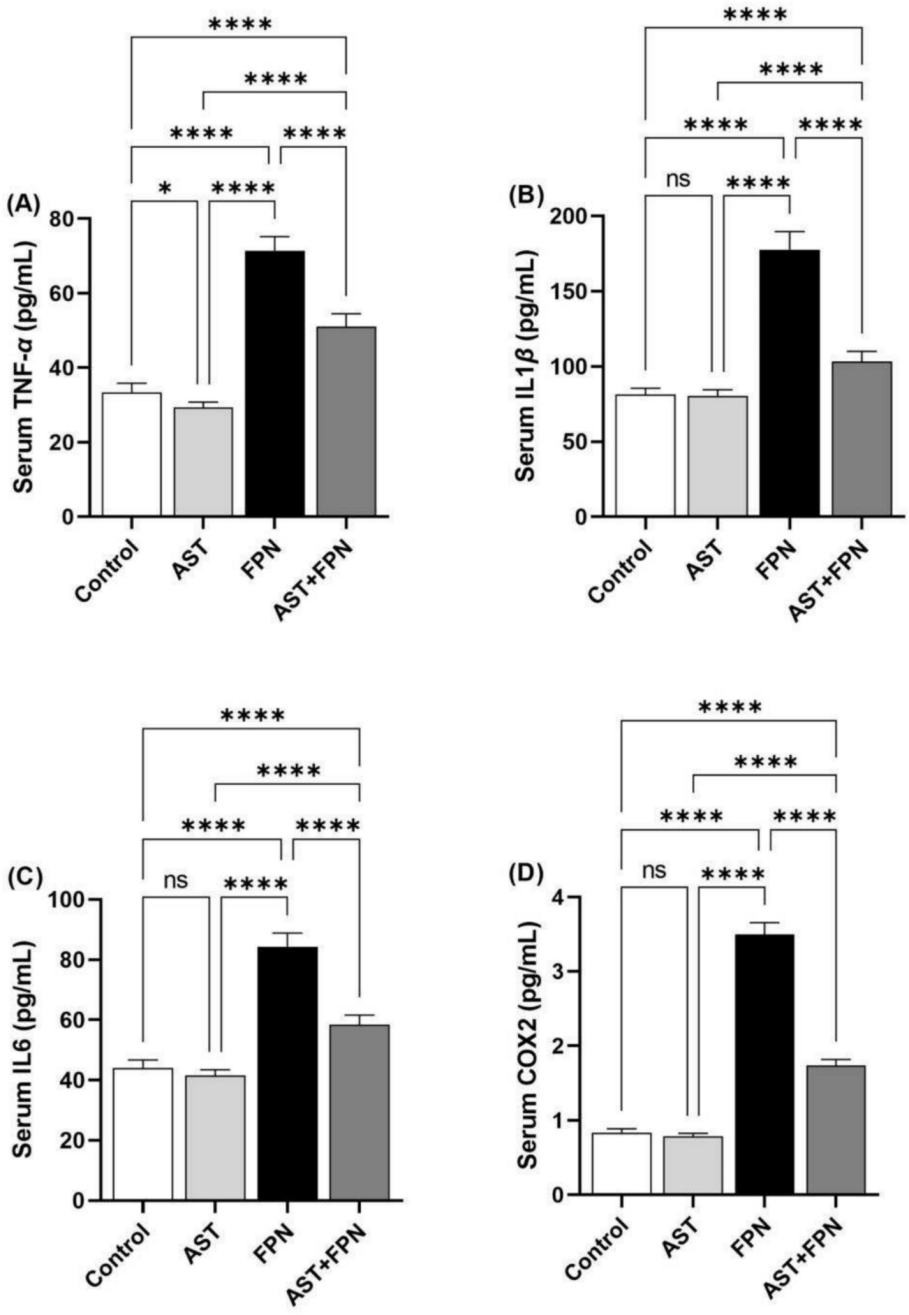
Effect of Astaxanthin (AST) (20 mg/kg bwt) on brain serum pro-inflammatory and inflammatory cytokines level of fipronil (FPN) (20 mg/kg bwt) administered male rats. (**A**) Tumor necrosis factor-*α* (TNF-*α*). (**B**) Interleukin-1*β* (IL-1*β*). (**C**) Interleukin-6 (IL-6). (**D**) Cyclooxygenase-2 (COX2). Data were analyzed with a one-way ANOVA followed by Tukey’s multiple comparison test. Data are expressed as the mean ± SD; n = 8. Means within columns carrying * are significantly different at *P* < 0.05 and ****P < 0.0001. ns = nonsignificant.

The pro-inflammatory and inflammatory biomarkers were evaluated to study the possible neuro-inflammatory impacts of FPN. The FPN group revealed a substantial (*P* < 0.001) increase in the serum content of IL-1*β*, TNF-*α*, IL-6, and COX2 compared with the control group. However, AST administration reduced the intensity of increased proinflammatory and inflammatory cytokines relative to FPN-administered rats (Fig. 3). Non-significant differences in the serum IL-1*β*, IL-6, and COX2 levels were found between AST-administrated and the control groups; in contrast, TNF-*α* was markedly (*P* < 0.05) decreased in the AST-supplemented rats relative to the control group.

### Histopathological examination and lesion scoring

Table 1 shows the semiquantitative evaluations of different groups’ histopathological lesions in the cerebral cortex and cerebellum. Normal histological limits were visible in both the control and AST-treated groups. However, tissue sections from FPN-treated rats showed substantial histopathological changes and a high grade in the recorded criteria. Conversely, co-administration of AST with FPN alleviated most of these changes, revealing mild to moderate pathological alterations.

**Table 1:** Lesions’ scoring in the cerebrum and cerebellum of the control, Astaxanthin (AST, 20 mg/kg bwt/day) and/or fipronil (FPN, 20 mg/kg bwt/day)-treated rats.

Tissue	Lesions	Control	AST	FPN	AST + FPN
1-**Cerebrum**					
	**Meninges**				
	-Vascular congestion	–	–	+ + +	+
	-Edema	–	–	+ +	+
	-Hemorrhage	–	–	+ +	+
	-Inflammatory cells infiltrations	–	–	+ +	+
	**Cerebral cortex**				
	-Degenerated neurons	–	–	+ + +	+ +
	-Necrotic neurons	–	–	+ +	+
	-Glial cells reactions	–	–	+ +	+
	-Neuropil vacuolation	–	–	+ +	+
	-Increased pericellular space	–	–	+ +	+
	-Perivascular cuffing	–	–	+ +	+
	-Vascular congestion	–	–	+ + +	+
	-Hemorrhages	–	–	+ +	-
2-**Cerebellum**					
	**Meninges**				
	-Vascular congestion	–	–	+ + +	+ +
	-Edema	–	–	+ +	+
	-Hemorrhage	–	–	+ + +	+
	-Inflammatory cells infiltrations	–	–	+ +	+
	**Cerebellar cortex**	–	–		
	-Pyknosis of Purkinje cells	–	–	+ + +	+ +
	-Increased pericellular space	–	–	+ +	+
	-Necrosis of Purkinje cells	–	–	+ +	+
	-Loss of Purkinje cells	–	–	+ +	+
	-Depletion of the granule cell layer	–	–	+ +	+
	**Cerebellar Medulla**	–	–	+ +	+
	-Gliosis	–	–	+	-
	-Vascular congestion	–	–	+ +	+
	-Hemorrhages	–	–	+ +	+

**Fig. 4:**
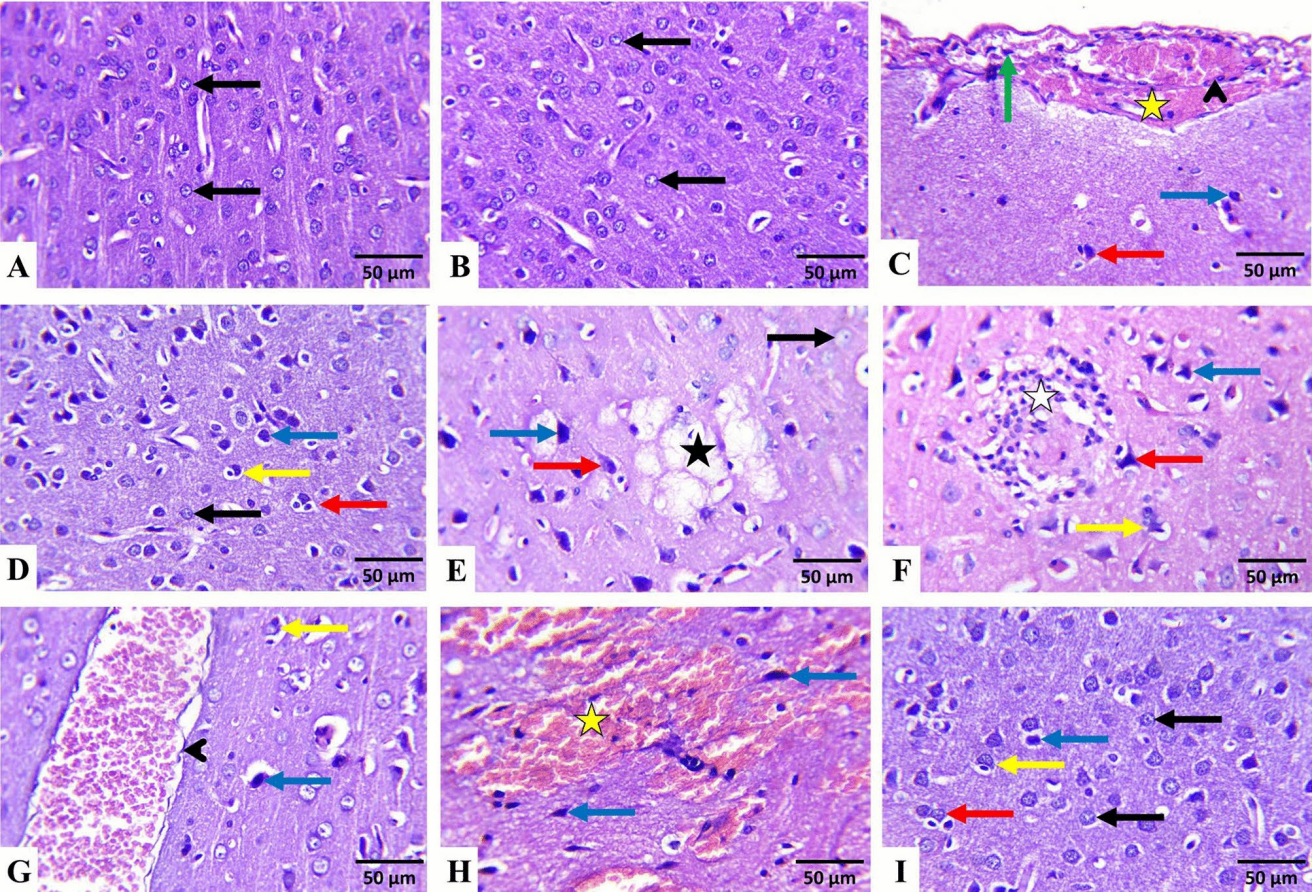
Representative photomicrographs of rats^,^ cerebrum (HE, × 400). (**A**) a control and (**B**) AST-treated rats showing normal histoarchitecture of the neurons (black arrows) and neuropil. (C–H) FPN-treated rats showing vascular congestion (arrowhead), inflammatory mononuclear cells infiltrations (green arrow), hemorrhage (yellow star), degenerated shrunken darkly stained neurons (blue arrow) and necrotic neurons associated with satellitosis (red arrow) and neuronophagia (yellow arrow), (**D**), varying sized vacuoles of the neuropil (black star) and malacic area with gliosis (white star). (I) AST + FPN- treated rat showing improvement of the cerebral tissue architecture with minimal degenerated darkly stained neurons (blue arrow) and necrotic neurons associated with satellitosis (red arrow) and neuronophagia (yellow arrow) as compared with normal neurons (black arrow).

**Fig. 5:**
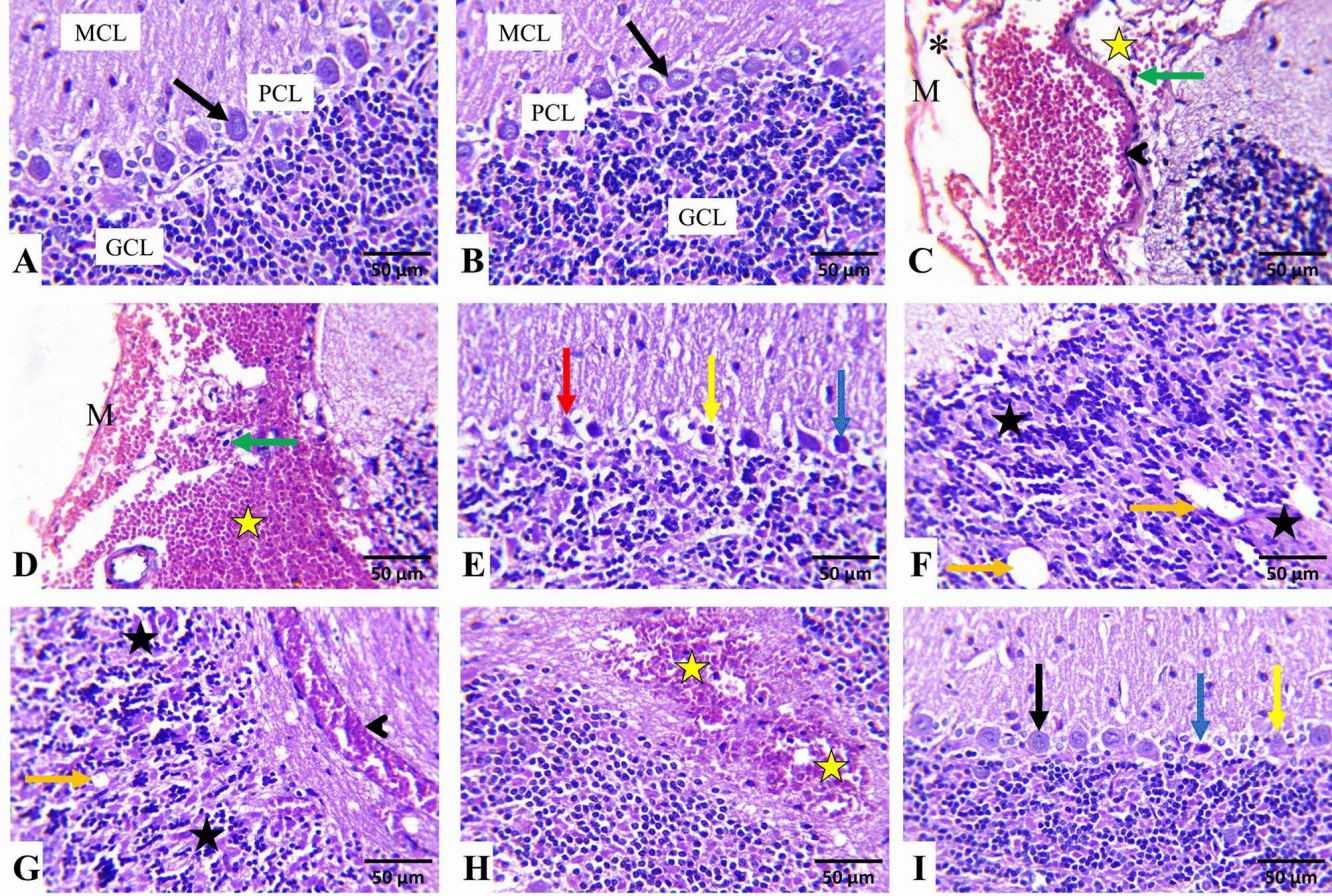
Representative photomicrographs of rats^,^ cerebellum (HE, × 400). (**A**) a control and (**B**) AST-treated rats showing normal histoarchitecture of molecular cell layer (MCL), Purkinje cell layer (PCL) with normal Purkinje cells (black arrow), and granule cell layer (GCL). (**C**–**H**) FPN-treated rats showing vascular congestion (arrowhead), inflammatory mononuclear cells infiltrations (green arrow), edema (*) and hemorrhage (yellow star) in the meninges (**M**), degenerated shrunken darkly stained Purkinje cells (blue arrow), and necrotic neurons associated with satellitosis (red arrow), and neuronophagia (yellow arrow), depletion of the granule cells (black star), hollow spaces in the granular cell layer (orange arrow), congestion (arrowhead) and hemorrhage (yellow star) in the cerebellar white matter. (**I**) AST + FPN-treated rat showing an improvement in the cerebellar tissue architecture with minimal degenerated darkly stained neurons (blue arrow) and necrotic neurons associated with neuronophagia (yellow arrow) as compared with normal neurons (black arrows).

The control and AST-treated rats had almost normal cerebral cortices (Fig. 4A,B, respectively) and cerebellar (Fig. 5A,B, respectively) histological features. By contrast, in FPN-treated rats, the cerebral tissues exhibited meningitis, as evidenced by vascular congestion, edema, hemorrhage, and inflammatory mononuclear cells infiltrations (Fig. 4C). Many cortical neurons were degenerated, shrunken, and deeply stained with increased pericellular spaces. Necrotic neurons exhibited pyknotic nuclei and hypereosinophilic cytoplasm, which may or may not be associated with satellitosis and neuronophagia (Fig. 4D). The neuropil had varying-sized vacuoles (Fig. 4E) and focal malacic areas with gliosis (Fig. 4F). Perivascular cuffing, congestion (Fig. 4G), and hemorrhages (Fig. 4H) were also perceived in most sections.

Concerning the cerebellar tissues of FPN-treated groups, the meninges displayed vascular congestion, edema (Fig. 5C), hemorrhage (Fig. 5D), and inflammatory mononuclear cells infiltrations. The cerebellar cortex showed a marked dissociation between the Purkinje cells layer and the other layers. Numerous shrunken Purkinje cells with pyknotic and hyperchromatic nuclei were visible (Fig. 5E). Others were necrotic and associated with satellitosis and neuronophagia with increased pericellular spaces. Also, there was a selective neuronal loss, and the granular cell layer showed cellular depletion and focal areas that showed hollow spaces (Fig. 5F). Additionally, focal areas of gliosis, congestion (Fig. 5G), and areas of hemorrhages (Fig. 5H) were evident in the cerebellar medulla.

Conversely, the cerebral (Fig. 4I) and cerebellar tissues (Fig. 5I) of AST + FPN-treated rats displayed improved histoarchitectures, with a considerable reduction in the severities and distributions of the earlier observed lesions (Table 1).

### Histomorphometrical analysis

**Fig. 6:**
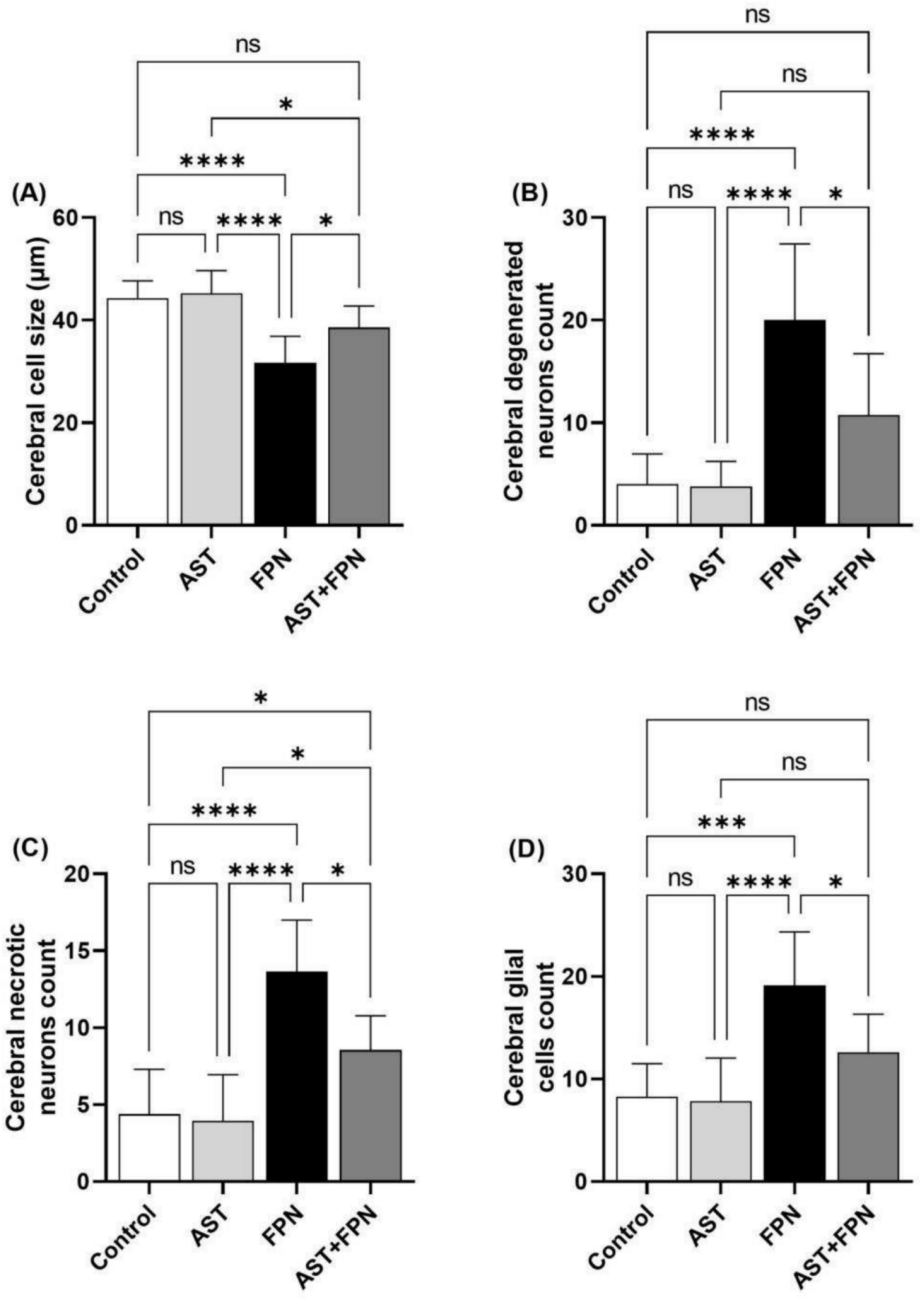
Histomorphometrical analysis of the cerebral cortices’ images of the experimental groups (images of H&E-stained sections, 10 different fields per section, HPF, × 400 for 8 rats per group). Data were analyzed with a one-way ANOVA followed by Tukey’s multiple comparison test. Data are expressed as the mean ± SD; n = 8. Means within columns carrying * are significantly different at *P* < 0.05, ***P < 0.001, and ****P < 0.0001. ns = nonsignificant. FPN, fipronil; AST, astaxanthin.

**Fig. 7:**
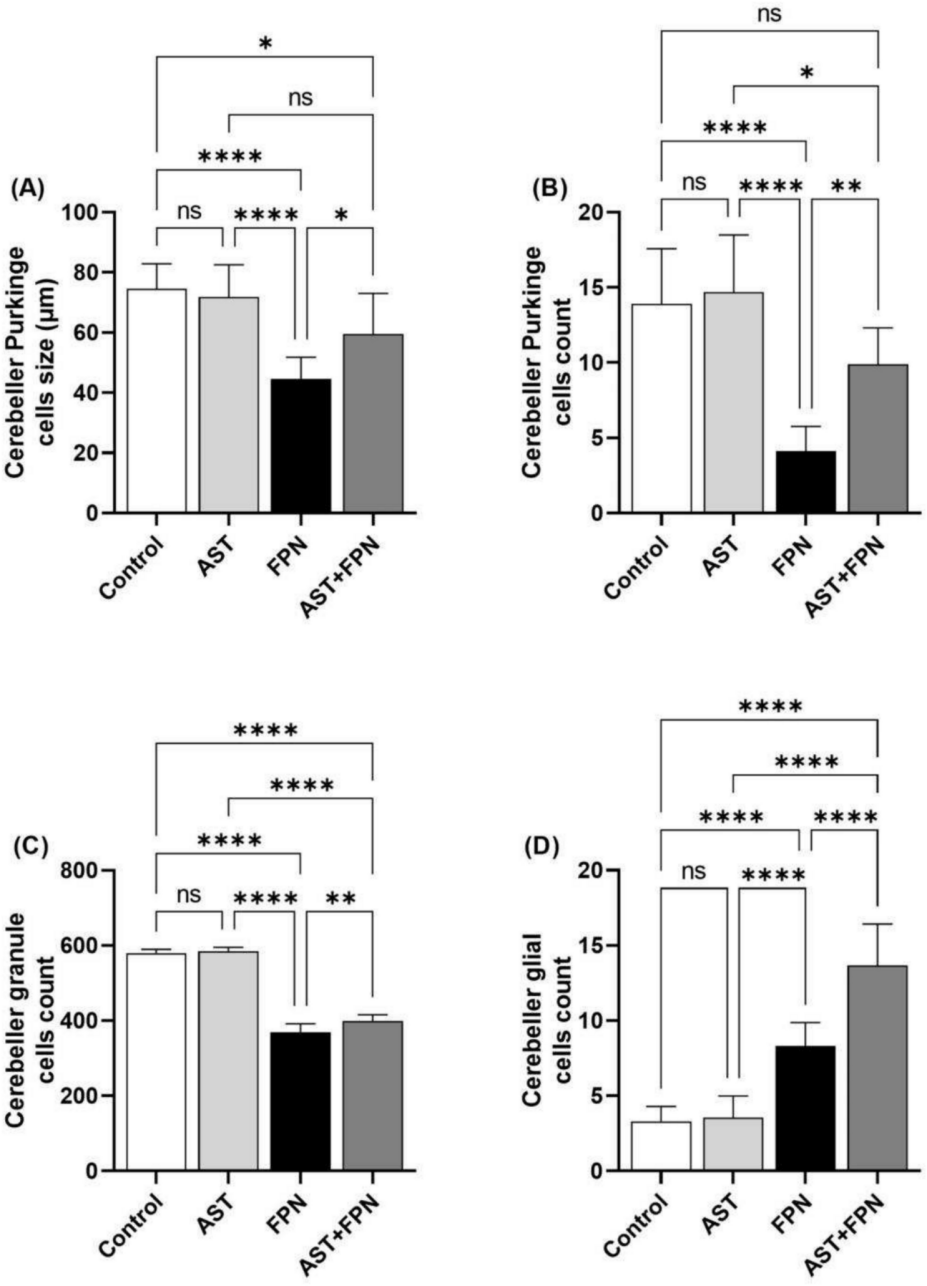
Histomorphometrical analysis of the cerebellar cortices’ images of the experimental groups (images of H&E-stained sections, 10 different fields per section, HPF, × 400 for 8 rats per group). Data were analyzed with a one-way ANOVA followed by Tukey’s multiple comparison test. Data are expressed as the mean ± SD; n = 8. Means within columns carrying * are significantly different at *P* < 0.05, **P < 0.01, and ****P < 0.0001. ns = nonsignificant. FPN, fipronil; AST, astaxanthin.

As demonstrated in Figs. 6 and 7, the image analysis of the control and AST-treated rat’s cerebral cortices showed no statistically significant changes in the mean values of the cerebral nerve cell size, degenerated and necrotic neuron counts, and mean glial cell count (Fig. 6A–D). Similarly, both groups exhibited no statistically significant differences in the mean values of the cerebellar Purkinje cell size, Purkinje, granule, and glial cell counts (Fig. 7A–D). Compared with the control group^,^s mean values, the FPN-treated group displayed a significant decrease in the mean cerebral neuron size and significant increases in the mean degenerated, necrotic, and glial cell counts (Fig. 6A–D). Also, the cerebellar tissues of this group exhibited significant decreases in the mean Purkinje cell size and count and the granule cell count. They showed a significant increase in the mean glial cell count (Fig. 7A–D).

On the contrary, the AST + FPN-treated group demonstrated a notable improvement of the previously mentioned parameters in the cerebrum and cerebellum compared with the FPN-treated group. Nevertheless, they were still comparable with the control group values (Fig. 6A-D and Fig. 7A–D).

### Immunohistochemical studies and quantitative assessment

**Fig. 8:**
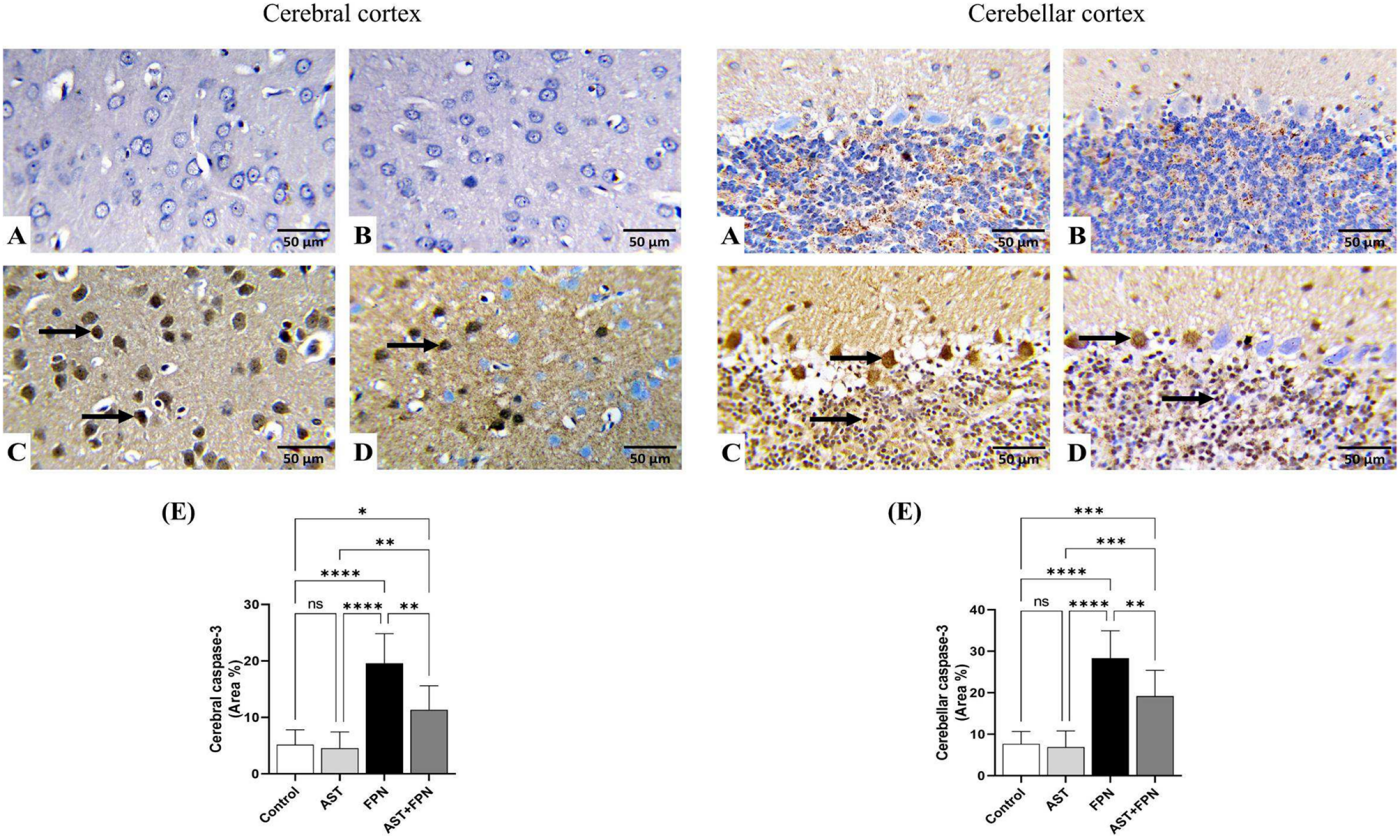
Representative photomicrographs demonstrating the immunohistochemical staining (brown staining) of cleaved caspase-3 in the cerebral and cerebellar tissue sections of the experimental groups (IHC, × 400). (**A**) Control, (**B**) Astaxanthin (AST), (**C**) Fipronil (FPN) and (**D**) AST + FPN- treated rats. (**E**) Quantification of cleaved caspase-3 expression, the immunohistochemical staining of cleaved caspase-3 was measured as the area percentage (%) across 10 different fields/section, n = 8 rat/group. Data were analyzed with a one-way ANOVA followed by Tukey’s multiple comparison test. Data are expressed as the mean ± SD; n = 8. Means within columns carrying * are significantly different at *P* < 0.05, **P < 0.01, ***P < 0.001, and ****P < 0.0001. ns = nonsignificant.

**Fig. 9:**
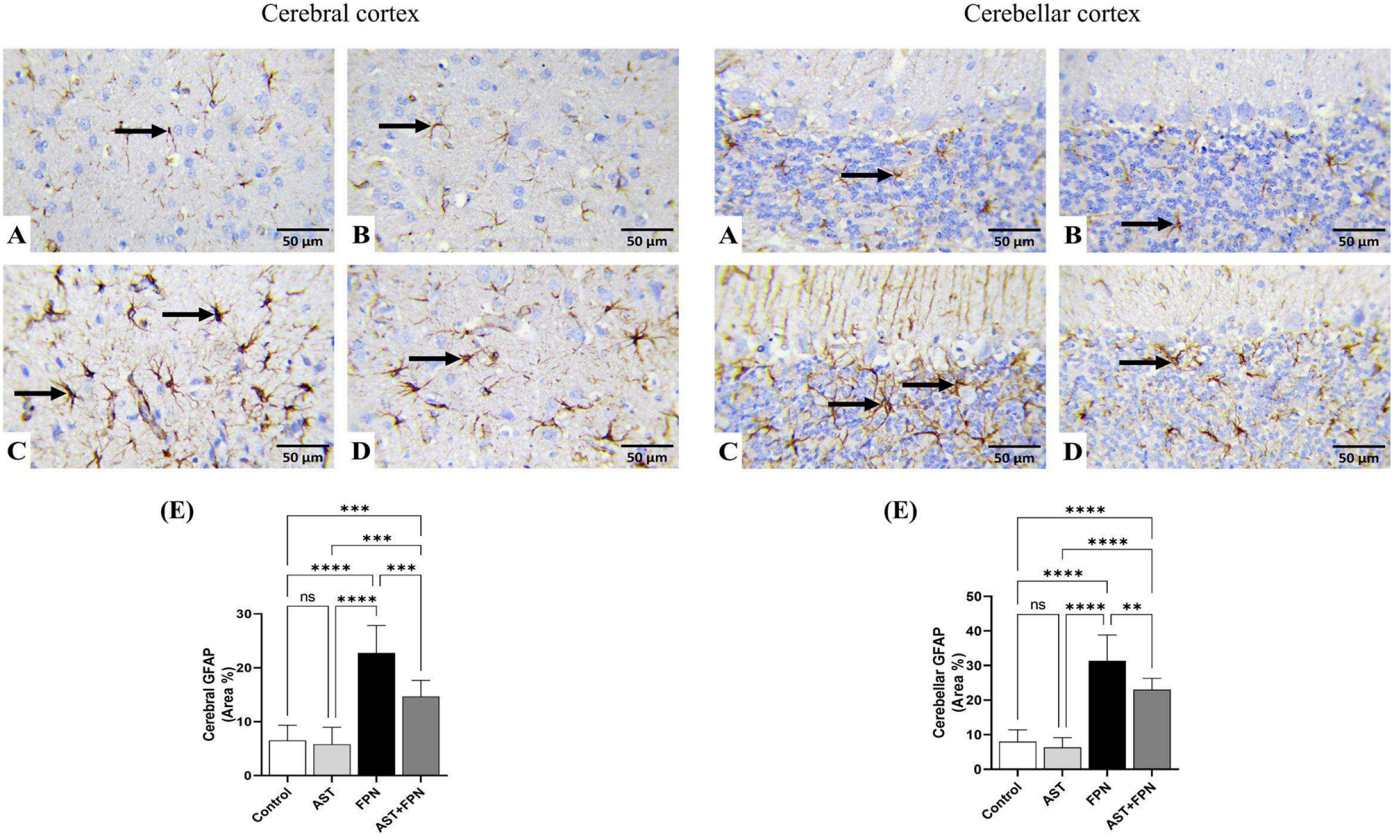
Representative photomicrographs demonstrating the immunohistochemical staining (brown staining) of the glial fibrillary acidic protein (GFAP) in the cerebral and cerebellar tissue sections of the experimental groups (IHC, × 400). (**A**) Control, (**B**) Astaxanthin (AST), (**C**) Fipronil (FPN) and (**D**) AST + FPN-treated rats. (**E**) Quantification of GFAP expression, the immunohistochemical staining of GFAP was measured as the area percentage (%) across 10 different fields/sections, n = 8 rat/group. Data were analyzed with a one-way ANOVA followed by Tukey’s multiple comparison test. Data are expressed as the mean ± SD; n = 8. Means within columns carrying ** are significantly different at *P* < 0.01, ***P < 0.001, and ****P < 0.0001. ns = nonsignificant.

Figures 8 and 9 exemplify the immunohistochemical staining of the cleaved caspase-3 and GFAP in the cerebral and cerebellar tissues and the quantitative assessment of their levels depending on the area percentages of positive immune-stained cells.

Concerning the cleaved caspase-3, the control (Fig. 8A) and AST-treated (Fig. 8B) groups revealed negative immunohistochemical staining of caspase-3 in the neurons and resident glial cells of the cerebral cortices and the Purkinje cells and granule cells in the cerebellar folia. There were no significant alterations in the mean area percentages of cleaved caspase-3 immunostained cells in AST-treated rats compared with the control group’s mean values (Fig. 8E).

The FPN-treated group (Fig. 8C) showed a strong positive immunoreactivity of cleaved caspase-3, as confirmed by prominent brown staining of the cerebral cortical neurons, Purkinje cells, and granule cells in the cerebellar folia. Relative to the control group^,^s mean values, the mean area percentages of cleaved caspase-3 positive immunostained cells showed significant increments in both cerebral and cerebellar tissues (Fig. 8E). Conversely, in AST + FPN-treated rats (Fig. 8D), most cerebral cortical neurons, cerebellar Purkinje, and granule cells exhibited weak to moderate immunoreactivity, and only a few cells showed intense immunopositive brown staining of cleaved caspase-3. Related to the FPN-treated group, the combination group showed significant decreases in the mean area percentages of cleaved caspase-3 positive immunostained cells in both cerebral and cerebellar tissues (Fig. 8E).

On the other hand, the immunohistochemical assessment showed that the GFAP immunoreactive-positive substances were observed in the astrocytes’ cell bodies and processes. The immunostained cells were widely distributed across the cerebral cortex and cerebellum in the control and the treated groups (Fig. 9). In the cerebral and cerebellum tissues of the control (Fig. 9A) and AST-treated (Fig. 9B) groups, the positive cells were brown, small, and had thin processes. The quantitative assessment showed no significant alterations in the GFAP immunostained cells’ mean area percentages between both groups (Fig. 9E). The FPN-treated rats had darker and relatively larger cells with thickened interdigitated processes in the examined tissues (Fig. 9C). Compared with the mean values of the control group, the FPN-treated group showed significant increments in the mean area percentages of GFAP-immunostained cells in both cerebral and cerebellar tissues (Fig. 9E). Meanwhile, in the AST + FPN-treated group, a substantial decrement in the astrocytic reaction was evident (Fig. 9D), with significant decreases in the mean area percentages of GFAP-immunostained cells in the cerebral and cerebellar tissues as compared with the FPN-treated group^,^s mean values (Fig. 9E).

### Molecular docking

**Fig. 10:**
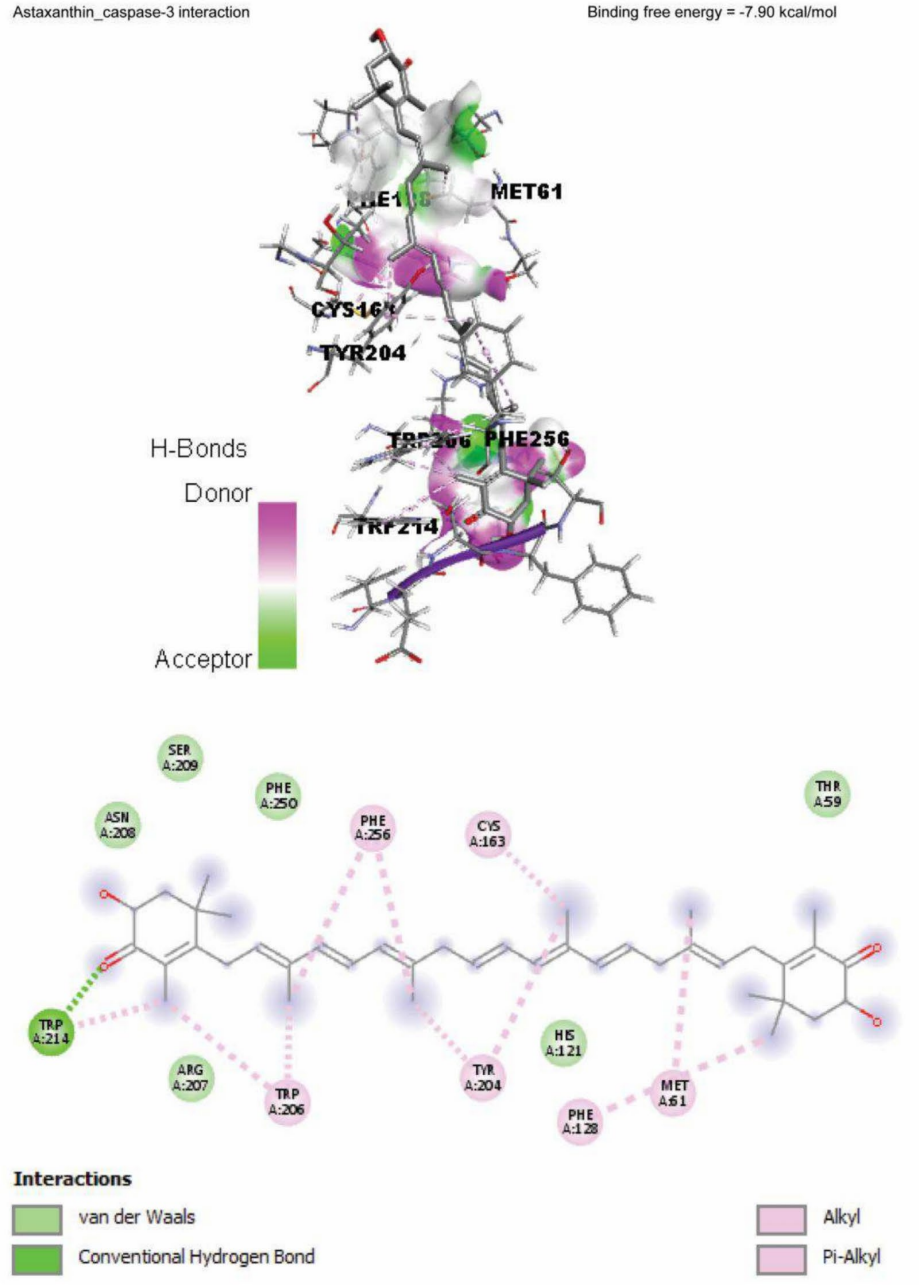
Molecular docking interaction of Astaxanthin (AST) active compounds with rats’ caspase-3.

**Fig. 11:**
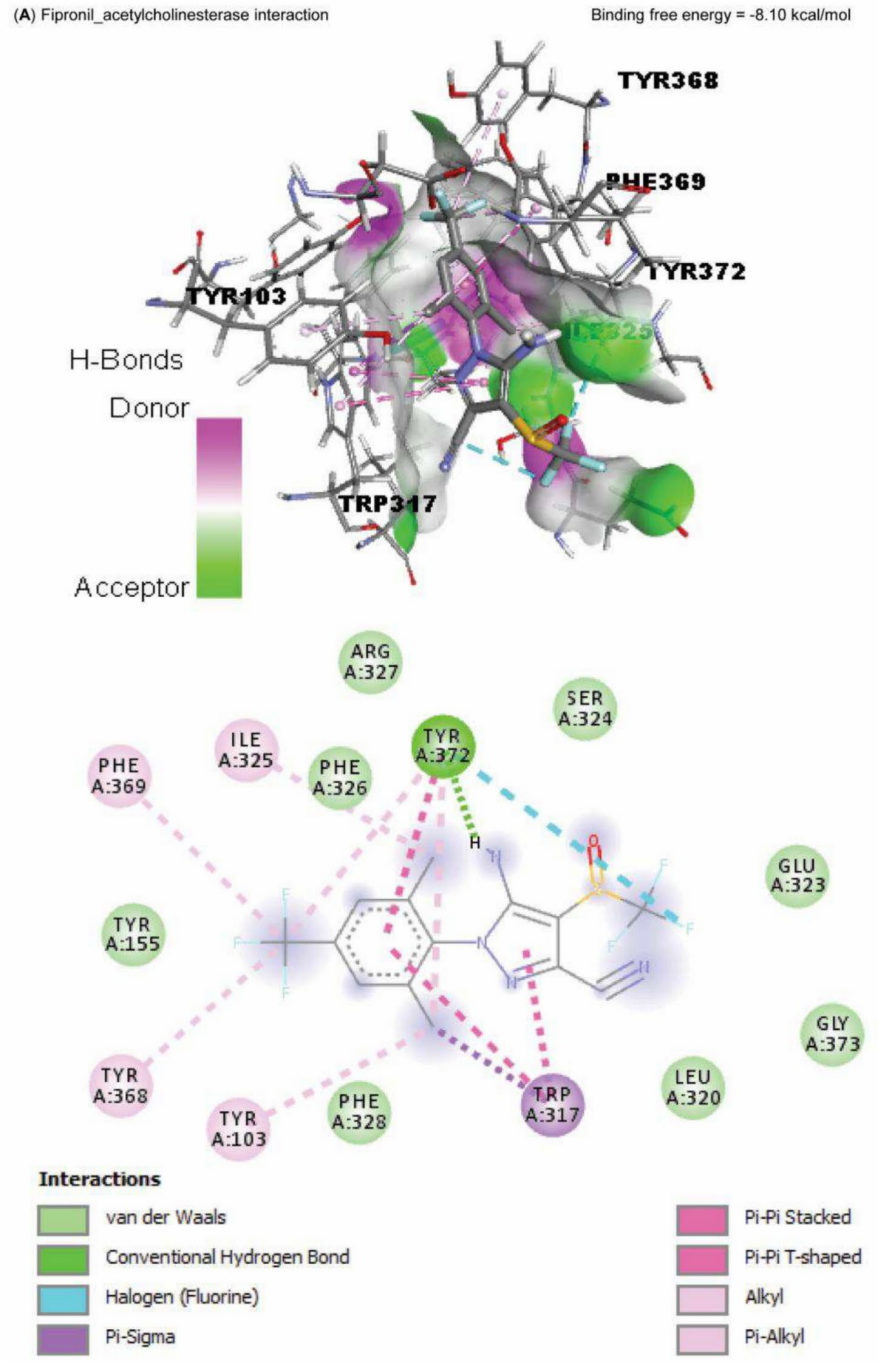

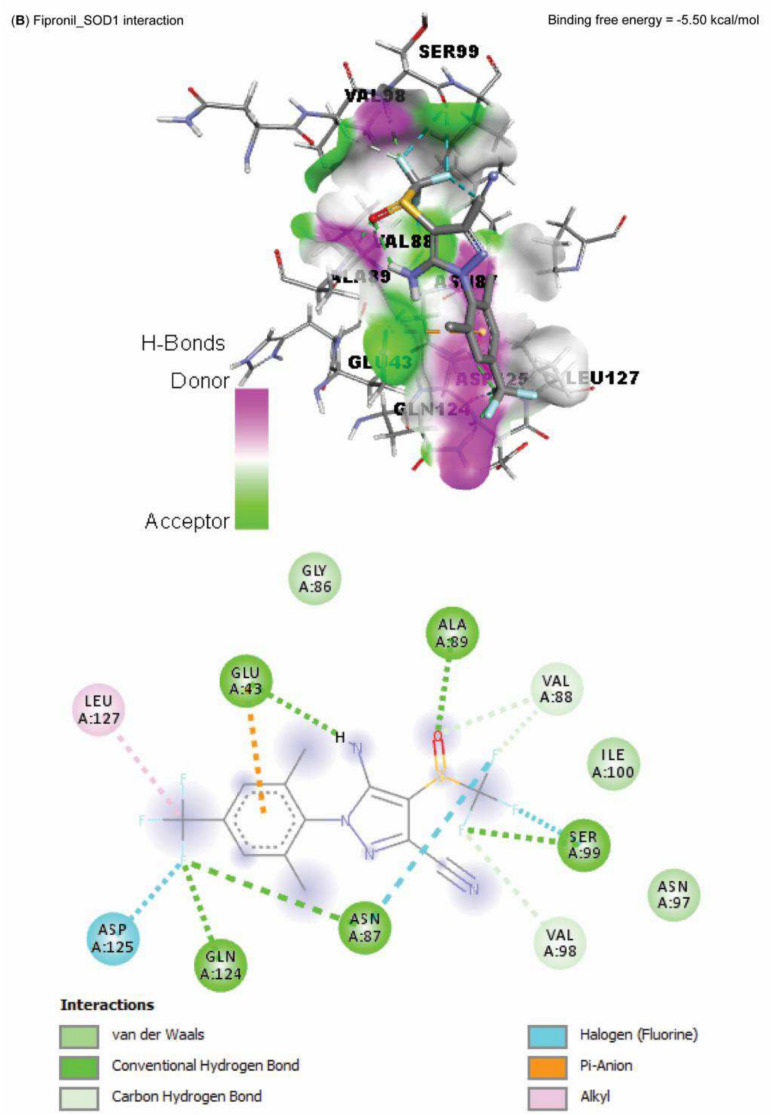

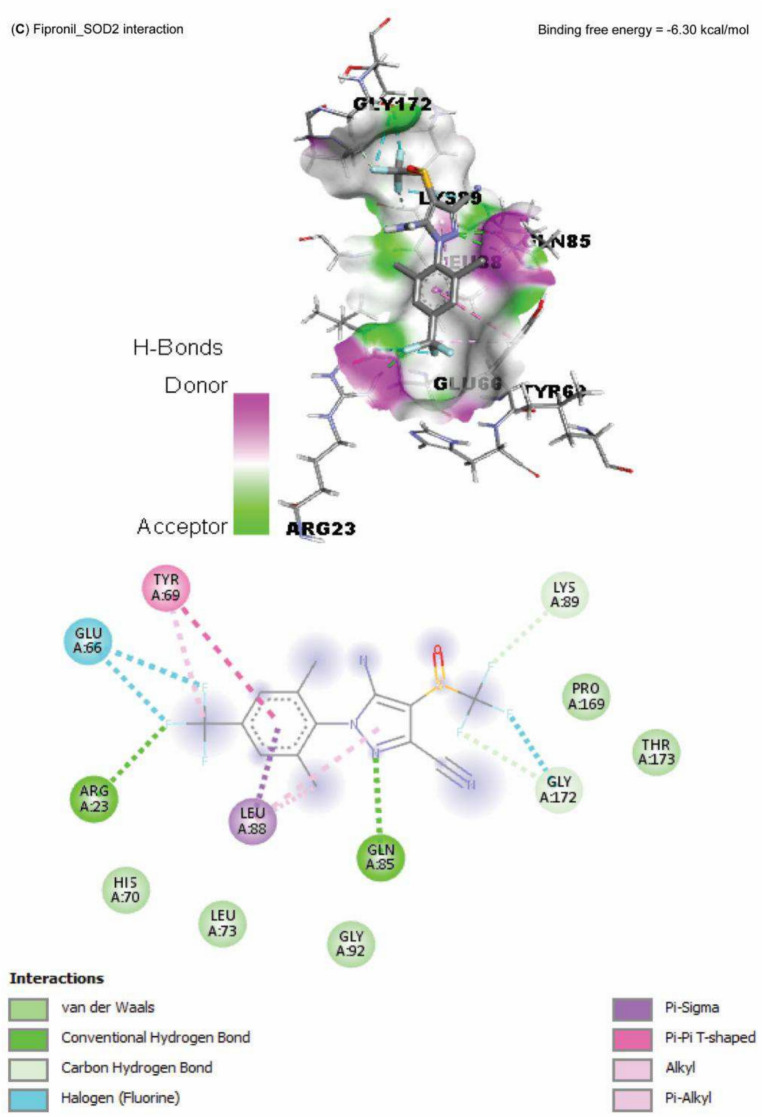

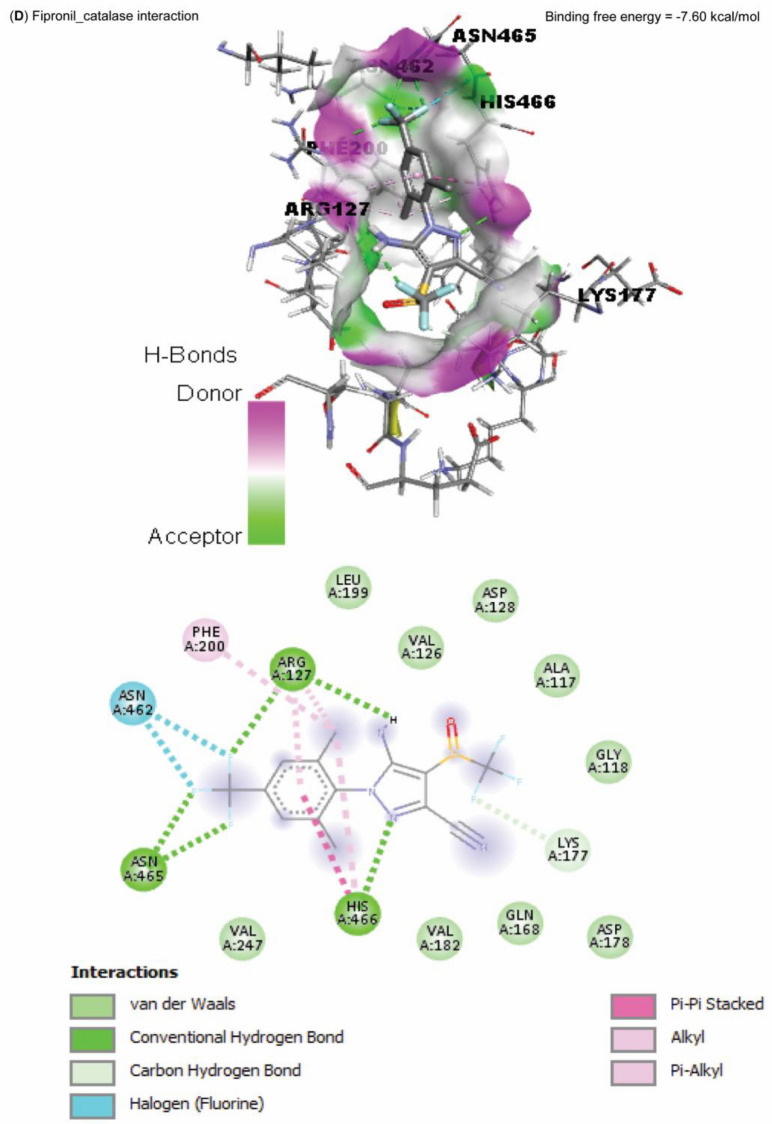
Molecular docking interaction of fipronil active compounds. Molecular docking interaction of fipronil with the binding site of rats’ (**A**) acetylcholinesterase, (**B**) superoxide dismutase-1 (SOD1), (**C**) superoxide dismutase-2 (SOD2), and (**D**) catalase.

Data represented in Fig. 10 explored the molecular interaction of AST and caspase-3, with a binding free energy of -7.90 kcal/mol. On the other hand, FPN interacted with the binding site of acetylcholinesterase, SOD1, SOD2, and CAT, with binding free energies of -8.10, -5.50, -6.30, -5.90, and -7.60 kcal/mol, respectively (Fig. 11A–E).

## Discussion

The brain, an essential organ in the body, serves as a system for coordinating and regulating all physiological functions^21,42^. The brain is a delicate tissue easily affected by ROS due to its elevated oxygen consumption, high peroxidizability of unsaturated fatty acids, and increased availability of highly peroxidizable substrates^13,18^. Furthermore, the antioxidant defenses of the brain are not very strong due to their limited capability to restore neurons that are more susceptible to pollutants^43^. The extensive use of pesticides in veterinary,  agricultural, and domestic settings poses a significant risk to target species, the environment, and any living things that encounter them^1,5^. Due to their high invertebrate toxicity, systemic nature, and ease of administration, phenylpyrazoles are among the most frequently used insecticides in the world^44^. This ensures that the insecticides are effectively disseminated throughout the treated region^10^. Pesticide effects on non-target creatures are thought to be the main component of effective pest management strategies. Pesticide exposure can have a wide range of harmful impacts on non-target organisms, such as nephrotoxicity, hepatotoxicity, neurotoxicity, hematotoxicity, etc., either directly or indirectly^9–11^. FPN is extensively consumed in rice farms to kill pest insects. Throughout the last decade, there has been a cumulative concern regarding the human and animal health and environmental impacts related to the consumption of FPN^45,46^.

To our knowledge, the neuroprotective effects of AST against FPN-induced neuronal damage have not been studied before. This study examined AST’s protective effects in adult male rats exposed to FPN. Male rats were chosen due to the physiological hormonal variations that is associated with the reproductive cycle of females, which may alter their brains’ neurological state and impact the measured parameters. In contrast, the male physiology is characterized by a particular stable hormonal variation, which is more suitable for the brain research experiments^2,3^. The current study found that FPN causes oxidative brain damage, disrupting cellular membrane functions and producing excess inflammatory cytokines. These cytokines may inhibit neurotransmitter synthesis and result in neuronal necrosis^47^. However, these adverse effects were mostly reversed when AST was administered with FPN. AST is a xanthophyll carotenoid found in algae, microbes, and marine life, especially crustaceans. It has properties that modulate the immune system, combat cancer, reduce inflammation, and act as an antioxidant^17^. Oxidative stress, caused by pollution, occurs when an imbalance between oxidants and antioxidants in cells increases free oxygen radicals^48,49^. According to earlier research, ROS are crucial for FPN-induced neuronal damage and death in both the *in-vitro* and *in-vivo* models^3,50^. The BBB permeability is compromised by oxidative stress^42^, which mediates mitochondrial damage, encourages oxidative damage, and exacerbates neuro-inflammation^51^. Because of the brain’s high oxygen consumption, high values of polyunsaturated fatty acids, and higher concentrations of iron and ascorbate that promote radical generation, the brain tissues are especially susceptible to oxidative stress^13^. It also has fewer antioxidants and antioxidant enzymes than other tissues^17^. Specific defensive mechanisms may be triggered by tissue damage resulting from ROS production. Antioxidants such as CAT, T-SOD, and GSH are the most critical defenders against free radicals, helping to inhibit lipid peroxidation^16,22^.

The brain has a higher energy demand than other tissues and contains many mitochondria to supply this energy. It is also susceptible to oxidative stress, and mitochondrial dysfunction can lead to issues with ATP production, which relates to neurodegenerative diseases^13,42^. Lipid peroxidation arises from damage to lipid structures in cell membranes and was studied in this investigation using MDA^52^. In our study, as predicted, brain MDA levels increased considerably in the FPN group relative to the control. The AST + FPN-treated rats showed a substantial decrease in MDA values in this experimental condition relative to the FPN group. An increase in MDA levels indicates increased oxidative stress and lipid peroxidation^20^. Our results are consistent with previous research findings on this topic^10,11,50^.

Hafez et al.^49^ reported that SOD is an enzyme that changes superoxide ions into H_2_O_2_. Furthermore, Nishida et al.^19^ stated that one of the most significant defense enzymes, CAT, transforms hydrogen peroxide into water and oxygen in all living things. Antioxidative indicators (GSH, T-SOD, and CAT) reveal an improvement in AST + FPN-treated rats compared with the FPN group and a reduction in the FPN group relative to the control. The current research findings agree with those of Khalaf et al.^2^ and Mahmoud et al.^3^ about the rat brain’s markedly decreased amounts of antioxidant enzymes caused by FPN.

The harmful impact of FPN on the antioxidant defense enzymes was also established by the molecular docking results of our study, which reported an interaction between FPN and the binding site of SOD1, SOD2, and CAT. The cellular antioxidant mechanism’s malfunction increases the cellular vulnerability to FPN-induced free-radical oxidation^10,11^. Furthermore, GSH eliminates free radicals such as singlet oxygen, superoxide, and hydroxyl radicals in all cells as the primary endogenous antioxidant^53^. FPN metabolites may cause a drop in GSH levels by preventing the cellular absorption of cysteine required for GSH production^9^. Conversely, in the AST + FPN group, T-SOD, GSH, and CAT activities were much higher than in FPN-administered rats; this could be explained by the antioxidative effects of AST and its ability to scavenge free radicals^17,20^.

All mammalian neurons have serotonin and dopamine, and alterations in their expression are associated with neurological diseases^20^. Dopamine regulates animal aggression, while serotonin generally inhibits brain activity depending on the receptor subtype it binds to^54^. The present investigation verified that rats in the FPN group had significantly enhanced brain dopamine and decreased brain serotonin levels. These might be related to the effects of FPN’s neuronal malfunction^55^. The data obtained agrees with the findings of Mahmoud et al.^3^, who reported reduced serotonin levels in the brains of rats given FPN. Tryptophan, an amino acid considered a precursor for serotonin production^56^, may interact with FPN, potentially reducing serotonin levels^54^. FPN primarily acts at the nerve terminal, inhibiting membrane fusion, limiting neurotransmitter release, and contributing to degeneration. This exact mechanism was previously explained by Abdel-Daim and Abdeen^27^. Additionally, the results showed that administering FPN dramatically raised dopamine, which may cause anxious behavior^11^. FPN’s neurotoxic effects on the brain’s antioxidant system and microglial cells may harm dopaminergic and serotonergic neurotransmission^57^. Oxidative stress and neuroinflammation can deteriorate one another, compromising the brain’s defenses and causing neuronal degeneration^52^. Increased inflammation caused by FPN may lead to the gradual damage of nigrostriatal dopaminergic neurons and S-2A receptors^8^. Impaired adult neurogenesis and neural maturation hinder neural replacement, deteriorating the condition and depleting serotonergic and dopaminergic neurotransmission^6^. These effects provide strong evidence of the neurotoxic effects of FPN insecticide on creatures that are not targets. According to the current study, AST reduced the neuronal damage caused by FPN by modifying the release of serotonin and dopamine in the brains of rats treated with AST + FPN, consistent with earlier research of Si and Zhu^21^.

Numerous research studies showed that fipronil sulfone was the cause of FPN-related neurotoxicity^9,11,50^, which is a mainly prevalent FPN metabolite in the livers of rodents and humans^27^. In mice and humans, fipronil sulfone has a six-fold greater affinity for GABA receptors than FPN^58^. The amino acid GABA neurotransmitter facilitates fast inhibitory neurotransmission in the brain^17^. At cholinergic synapses, neurotransmission is stopped by the swift enzyme AchE^56^. Additionally, AchE is thought to be a possible biomarker of neuronal damage and is essential for transmitting neuromuscular impulses^18^. An increase in oxidative damage is frequently linked to AchE activity. The capacity of AchE to neutralize ROS may be responsible for its neuroprotective effects^20^. This study’s findings that FPN inhibited AchE activity are in line with those of Mahmoud et al.^3^, who found that FPN toxicity results in AchE reduction and that increased ACh appearance leads to increased ROS and inflammation. In addition, the molecular docking findings of our study revealed the interaction between FPN and the binding site of AchE, which confirmed the inhibition of AchE activity and the further reduction of its functions. GABA is an inhibitory neurotransmitter that calms when it binds to a GABA receptor^24^. According to this study, FPN significantly decreased GABA levels, indicating that the neuroendocrine mechanism that caused the neuronal damage caused by FPN was dysregulated^57^. Akkoyun et al.^21^ supported this study by showing that AST has a robust anxiolytic function facilitated via the GABAergic neurons. Our findings suggest modulating the oxidative/antioxidant balance positively affects neurotransmitter regulation. This improvement may enhance neurotransmitter production, release, and storage, reducing degenerative brain lesions. Thus, AST may offer neural protection against FPN through these mechanisms^16,17,20^.

Consistent with our findings, it was demonstrated that FPN treatment for four weeks raised neuroinflammatory markers^2,3,11^. It has been shown that FPN increases oxidative stress and triggers inflammatory cascades, leading to neuronal dysfunction^9,27^. TNF-*α*, IL-1*β*, IL-6, and COX2 have all been demonstrated to rise in response to FPN poisoning in the tissues of the hippocampus^50^. These increased levels could result from inflammation in the brain and neuronal damage^48^. FPN caused hippocampal microglia and astrocytes to proliferate and become more active, potentially increasing inflammatory cytokine expression^14^, such as IL-6, TNF-*α*, and IL-1*β*, which could exacerbate oxidative stress and inflammation^3^. TNF-*α* induced inducible nitric oxide synthase and peroxynitrite intracellular expression, which resulted in either neuronal death or a change in synaptic plasticity^57^. Following brain injury, COX2 activation results in increased prostaglandin E2 (PGE2) levels^59^. Since IL-6 can cross the BBB, it may also contribute to producing PGE2^22,60^. Consequently, a positive relationship exists between elevated PGE2 and elevated values of IL-6 and COX2. These are likely reasons for the observed neuronal dysfunction after 4 weeks of FPN treatment. Conversely, the AST + FPN group’s IL-1*β*, IL-6, and TNF-*α* levels returned to normal following AST treatment, possibly related to AST’s anti-inflammatory properties^16,25,61^.

The histopathological, histomorphometrical, and immunohistochemical findings were consistent with the biochemical results. In the FPN-treated group, we observed various histopathological alterations, including neuronal degeneration, apoptosis, necrosis, glial cells reactions, and increased pericellular spaces in the cerebral and cerebellar tissues. These changes were likely a result of FPN-induced oxidative stress, generation of ROS, mitochondrial dysfunction, and cytoskeleton disruption, which consequently led to cell death^62,63^. The ROS disrupt essential cellular processes by interacting with DNA, RNA, and protein synthesis, which may result in inflammation, cell necrosis, or apoptosis^14^. They also target polyunsaturated fatty acids in cell membranes, leading to membrane breakdown and cellular vacuolization^64^. Additionally, ROS can disrupt brain vascular function, explaining the edema and hemorrhages found in the neural tissues with eventual cell damage and death^65^. The reported cerebral and cerebellar lesions were aligned with those stated by Mahmoud et al.^3^, Awad et al.^63^, Abou-Zeid et al.^66^, and Bakr et al.^55^. Conversely, the concurrent administration of AST with FPN partially ameliorated these histopathological findings, as the histopathological scoring shows. This effect was likely due to AST’s ability to cross the BBB, scavenge free radicals, and alleviate FPN-induced oxidative stress^17^. So, it has the potential to treat neurological diseases and mitigate the histological changes which occurred in the brain tissue^21^, as reported in previous studies such as the mouse model of vascular cognitive impairment induced by repeated cerebral ischemia/reperfusion injury^45^, acute cerebral infarction in rats^67^, traumatic brain injury in mice^68^  and many neurodegenerative disorders, including Alzheimer’s^69,70^, Parkinson’s^71^, and autoimmune diseases^72^ in which oxidative stress mechanisms play a significant role in the disease pathogenesis^26,73^.

On the other hand, the histomorphometrical analysis indicated that compared with the control group's mean values, the cerebral cortices of FPN-treated rats revealed a substantial reduction in mean nerve cell size in addition to significant increases in the mean degenerated and necrotic neuron and glial cells^,^ counts. Furthermore, the cerebellar cortex of this group exhibited significant decreases in the mean Purkinje cells’ size and count and the granule cells’ count, as well as a significant increase in the mean glial cells’ count. These results may be related to oxidative damage, neuronal inflammation, necrosis, and progressive loss of neurons^48,74^. Contrariwise, the simultaneous administration of AST with FPN partially improved these alterations. These effects could be attributed to the ability of AST to mitigate oxidative damage, reduce lipid peroxidation and inflammation, and maintain the cell’s integrity^73^.

Apoptosis, or programmed cell death, is a cellular mechanism that eliminates injured and infected cells or those that have reached the end of their lifespan^75^. It involves the expression of specific genes and the regulation of several proteins^76^ including anti-apoptotic protein B cell lymphoma 2 family proteins, which are mainly located in the mitochondrial membrane and play an essential role in reducing apoptosis^77^ and apoptotic genes, including caspases^78^. The detection of active caspase-3 offers a valuable and accurate way to identify apoptotic cells in tissues before all morphological signs of apoptosis appear^79^. There are many diseases and neurodegenerative disorders characterized by the upregulation of caspases^80^. Since FPN and its metabolites can cross BBB and the neuronal membrane^81,82^. They provoke the production of ROS, which mediate mitochondrial injury and interfere with the permeability of the mitochondria^7^ leading to the production of key apoptotic proteins, including caspase-3 and cytochrome-c^44^.

In the current experiment, the immunohistochemical examination displayed that the FPN-treated group showed a strong positive immunoreactivity of cleaved caspase-3, as confirmed by prominent brown staining of the cerebral cortical neurons, Purkinje, and granule cells in the cerebellar folia. These findings were affirmed by quantitative analysis, which demonstrated substantial increases in the mean area percentages of cleaved caspase-3 positive immunostained cells in the examined tissues. Our results followed those reported by Awad et al.^63^ and Elshony et al.^57^, which proposed that FPN triggered the apoptotic signal via mitochondrial damage and upregulation of caspase-3 expression.

Concurrent treatment of AST with FPN modulated the cleaved caspase-3 immunohistochemical staining in the examined tissues, as most cerebral cortical neurons, cerebellar Purkinje, and granule cells exhibited weak to moderate immunoreactivity. In contrast, only a few cells displayed intense immunopositive brown staining of cleaved caspase-3 with substantial decreases in the mean area percentages of cleaved caspase-3 positive immunostained cells in both cerebral and cerebellar tissues. The immunohistochemical findings were consistent with the molecular docking findings of our study. The anti-apoptotic activity of AST could be due to its anti-inflammatory and antioxidant properties, as recorded by Fang et al.^83^ and Zhang et al.^84^.

Astrocytes, the most prevalent cells in neural tissue, respond to central nervous system injuries through reactive astrogliosis. It is considered a key marker of structural changes in CNS. In reactive astrogliosis, astroglia cells experience cellular hypertrophy (increased size and GFAP protein expression) and hyperplasia (increased number of glial cells)^85,86^. FPN’s overproduction of ROS may trigger the progression of reactive astrogliosis and the corresponding inflammatory and apoptotic reactions. GFAP, a specific marker for mature astrocytes, is astrocytes’ key intermediate filament III protein. It is essential for maintaining the cytoskeleton structure and mechanical strength of astrocytic processes and for supporting neighboring neurons. GFAP corroborates BBB integrity, white matter architecture, and myelination^85^. Furthermore, it preserves the structural integrity of astrocytes, especially when these cells experience hypertrophy and hyperplasia in response to CNS damage, which is associated with neuroinflammation and neurodegenerative diseases. GFAP serves as an early and sensitive biomarker of neurotoxicity, and its upregulation precedes the perceptible histological alterations in the brain^87,88^.

GFAP-immunoreactive substances were observed in the cell bodies and processes of the astrocytes across astrocytes of the cerebral cortex and cerebellum. In the FPN-treated group, we observed darker, larger cells with thickened interdigitated processes and a significant increase in the mean area percentages of GFAP-positive immunostained cells in cerebral and cerebellar tissues relative to the control rats. This showed that FPN was linked to brain injury and neuroinflammation. These findings were agreed with those recorded by Awad et al.^63^.

The co-administration of AST with FPN substantially decreased the astrocytic reaction, evident in the significant decreases in the mean area percentages of GFAP in cerebral and cerebellar tissues relative to FPN-administered rats’ mean values. The neuroprotective effect of AST could be explained by its proven capability to alleviate oxidative stress, improve resistance to the free radicals^,^ attacks^20^  that were triggered by FPN and to boost the total antioxidant defenses and regulation of redox homeostasis^89^, which in turn limited oxidative stress-driven neuroinflammation and modulated the immunohistochemical staining of the GFAP, as reported by Ying et al.^46^.

## Conclusion

AST emerges as a promising therapeutic option in countering the oxidative stress and inflammatory responses triggered by FPN exposure in the rat brain. This study marks a significant milestone, revealing for the first time that AST can substantially alleviate the neuronal damage caused by FPN. Although the precise mechanisms through which AST exerts its protective effects remain uncertain, it is plausible to surmise that its neuromodulatory properties play a crucial role. Specifically, AST appears to influence the balance of neurotransmitters, modulate oxidative stress levels, and impact the expression of pro-inflammatory cytokines and the markers indicative of neuronal apoptosis, such as cleaved caspase-3 and astrogliosis as GFAP which were evident through immunohistochemical staining. Despite these encouraging findings, further investigation at the molecular level is essential to fully elucidate the intricate pathways through which AST mitigates FPN-induced neuronal injury.

## Data Availability

Data will be made available on reasonable request from the corresponding author (M.H.H.)

## Abbreviations

5-HT: 5-Hydroxytryptamine
AchE: Acetylcholinesterase
AST: Astaxanthin
BBB: Blood–brain barrier
CAT: Catalase
COX2: Cyclooxygenase-2
DAB: 3,3-diaminobenzidine tetrahydrochloride
DMSO: Dimethyl sulfoxide
DPX: Di-poly cysteine xylene
EDTA: Ethylenediamine tetra-acetic acid
FPN: Fipronil
GABA: Gamma-aminobutyric acid
GFAP: Glial fibrillary acidic protein
GSH: Reduced glutathione
IL-1*β*: Interleukin-1*β*
IL-6: Interleukin-6
MDA: Malondialdehyde
ROS: Reactive oxygen species
TNF-*α*: Tumor necrosis factor-*α*
T-SOD: Total superoxide dismutase
